# Streamlining the Detection of Human Thyroid Receptor Ligand Interactions with XL1-Blue Cell-Free Protein Synthesis and Beta-Galactosidase Fusion Protein Biosensors

**DOI:** 10.3390/life13101972

**Published:** 2023-09-27

**Authors:** J. Porter Hunt, Tyler J. Free, Jackelyn Galiardi, Kevin M. Watt, David W. Wood, Bradley C. Bundy

**Affiliations:** 1 Department of Chemical Engineering, Brigham Young University, Provo, UT 84602, USA; 2 Department of Chemical and Biomolecular Engineering, The Ohio State University, Columbus, OH 43210, USA; 3 Department of Pediatrics, University of Utah, Salt Lake City, UT 84108, USA

**Keywords:** cell-free protein synthesis, thyroid receptor ligand, fusion protein, reporter enzyme

## Abstract

Thyroid receptor signaling controls major physiological processes and disrupted signaling can cause severe disorders that negatively impact human life. Consequently, methods to detect thyroid receptor ligands are of great toxicologic and pharmacologic importance. Previously, we reported thyroid receptor ligand detection with cell-free protein synthesis of a chimeric fusion protein composed of the human thyroid receptor beta (hTRβ) receptor activator and a β-lactamase reporter. Here, we report a 60% reduction in sensing cost by reengineering the chimeric fusion protein biosensor to include a reporter system composed of either the full-length beta galactosidase (β-gal), the alpha fragment of β-gal (β-gal-α), or a split alpha fragment of the β-gal (split β-gal-α). These biosensor constructs are deployed using *E. coli* XL1-Blue cell extract to (1) avoid the β-gal background activity abundant in BL21 cell extract and (2) facilitate β-gal complementation reporter activity to detect human thyroid receptor ligands. These results constitute a promising platform for high throughput screening and potentially the portable detection of human thyroid receptor ligands.

## 1. Introduction

Delicate and intricate biological pathways must be precisely regulated to sustain human life. Thyroid receptor signaling controls major physiological processes and disrupted signaling can cause severe disease, including cancer and metabolic and neurological disorders [1,2,3]. Inadvertent exposure to endocrine disrupting chemicals is a health hazard [4,5], while thyroid receptor ligands are an important and emerging class of pharmaceuticals used in medical interventions to treat a variety of health conditions [6]. Consequently, methods to detect thyroid receptor ligands are of great toxicologic and pharmacologic interest.

**Figure 1:**
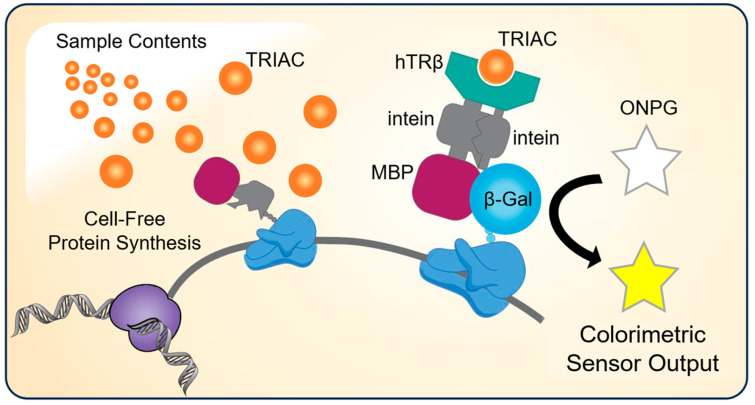
Cell-free protein synthesis of a chimeric fusion protein in the presence of a sample containing thyroid receptor ligand (e.g., TRIAC) results in a colorimetric sensor output. The β-gal biosensor construct developed in this work is composed of the following domains: maltose binding protein (MBP), intein, human thyroid receptor beta (hTRβ), intein, and a reporter domain (β-gal). The protein translated in the presence of receptor ligand results in the activation of the reporter enzyme to produce a colorimetric signal output.

Ligand detection strategies that employ human receptor proteins can be effective for disruptor compound screenings [7,8]. Recently we reported the utility of a chimeric fusion protein containing a human thyroid receptor domain (hTRβ) fused to a beta lactamase (β-lac) reporter [9]. This fusion protein only detects ligands during protein folding and thus must be translated in the presence of the ligand or sample [10], as depicted in Figure 1. Therefore, this fusion protein was expressed using a BL21 *E. coli*-based cell-free protein synthesis (CFPS) system that enabled the rapid detection of human thyroid receptor ligands with a β-lac/nitrocefin colorimetric enzymatic readout [9,10,11]. However, the relatively high cost and poor stability of nitrocefin [12,13,14] motivate efforts to reengineer the fusion protein with alternative reporter enzyme systems. The cell-free protein synthesis biosensing reactions prepared with crude extract systems cost USD 0.28 per sensing assay (70 μL in this work) [15] and the nitrocefin substrate costs USD 0.43 per sensing assay (0.2 mL [9]) which is 60% of the total cost of USD 0.71 per assay.

To reduce the biosensor costs, the relatively expensive β-lac/nitrocefin reporter system needed to be replaced with a different reporter system, such as one that has already facilitated CFPS biosensing [16,17,18]. Selecting a reporter protein for a given application involves considerations of cost, sensitivity, output visibility, and other factors. For this work, reporter systems with low cost and high visibility outputs were prioritized. The β-lac reporter and other enzymes hydrolyze a colorimetric substrate to provide a visual sensor output [9], while the NanoLuc enzyme generates a luminescent signal from substrate hydrolysis [17,19]. Other types of reporter enzymes hydrolyze substrates that can be detected electronically or by other methods, which is the case with invertase and a personal glucose meter readout [20]. As a simpler alternative, fluorescent proteins such as sfGFP do not require enzymatic reactions and thereby avoid potentially expensive substrates [21,22,23]. Furthermore, fluorescent protein sensitivity was comparable to other reporters in a controlled study using spectrophotometric equipment [18]. However, enzymatic reporters such as β-galactosidase (β-gal) are often preferable to fluorescent proteins for visual signal recognition due to their vibrantly colored substrates [23]. 

In this work, the β-lac/nitrocefin reporter system was replaced with β-gal and the substrate o-nitrophenyl-β-D-galactopyranoside (ONPG) which reduces the substrate cost by 98% and reduces the total biosensor cost to USD 0.29 per assay, which is a 60% reduction in total biosensor reagent cost (Figure S1). To leverage the cost savings of the ONPG assay substrate, the biosensor construct is reengineered in this work with the β-gal reporter enzyme. The β-gal reporter system has been extensively studied, and multiple variations have been used in whole-cell [24] and cell-free sensing applications, including split versions [25]. In this work, three novel chimeric fusion protein biosensors were created to enact three distinct reporter enzyme strategies using either full-length β-gal, the alpha-fragment of β-gal (β-gal-α), or a spliced alpha-fragment of β-gal (split β-gal-α) [26,27,28,29,30,31,32]. To utilize the β-gal reporter constructs, endogenous β-gal activity must be eliminated from the cell extract source strain [24]. Furthermore, to utilize split versions of β-gal, a source of β-gal omega fragment must be present for complementation. The *E. coli* XL1-Blue strain has been used to accomplish alpha complementation for in vivo sensing [24], and this work presents the novel use of cell extract made from the *E. coli* XL1-Blue strain to accomplish both objectives in the cell-free biosensing system. Each β-gal construct successfully demonstrates an increasing colorimetric signal with increasing thyroid receptor ligand concentration.

Biosensors capable of detecting endocrine disrupting compounds (EDC) are essential tools for reducing the enormous burden on human health and the economy that is caused by the inadvertent exposure to EDCs. Prostate cancer, breast cancer, infertility, reproductive dysfunction, birth defects, obesity, diabetes, cardiopulmonary disease, and neurobehavioral and learning dysfunctions have all been linked to EDC exposure [33,34]. Specific examples of exposure consequences are known in many instances. Polybrominated diphenyl ethers (PDBE) cause neurobehavioral dysfunction, phthalates are strongly associated with early cardiovascular deaths, bisphenol A is associated with childhood obesity, and dichlorodiphenyltrichloroethane exposure is associated with diabetes [34]. These and other disease-related implications of EDC exposure incur a tremendous economic burden on society. While the true costs of the cumulative direct and indirect exposure of EDCs will never be known, statistics and probability can be useful to provide an economic burden estimate. According to a study by Attina et al., the United States economic disease burden is estimated at USD 340 billion, which is over 2% of the USA gross domestic product (GDP) for the year 2016 [34]. The European Union disease burden is estimated at 1% of GDP, and the authors of the study explain that the chemical regulatory differences likely account for a large portion of the difference, suggesting that proactive prevention and improved screening for EDCs could dramatically reduce the USD 340 billion annual disease burden [34]. Estrogen and thyroid receptors are among the critical types of endocrine receptors which are vulnerable to disrupted signaling caused by EDCs, which has prompted our prior work to develop sensors for ligands that interfere with either receptor [9,11], and the thyroid receptor ligand biosensor construct is the focus of the engineering efforts in this work. 

Many different natural and synthetic molecules can act as ligands to signal thyroid hormone receptors by inducing conformation changes in the human thyroid receptor beta (hTRβ), including more than 150 documented chemicals and many more uncharacterized compounds [33,35]. For toxicological screening and pharmaceutical dosage purposes, the cumulative hormone binding activity of a sample is an important metric, even if all the individual contributing species are not distinctly identified [36,37]. Previous embodiments of the hTRβ biosensor featured in this work have been demonstrated with sensitivity to T3 and 3,3′,5-triiodothyroacetic acid (TRIAC) [9]. TRIAC, a human thyroid receptor ligand, was used in this work to assess the performance of newly presented hTRβ biosensors. TRIAC is a clinical therapeutic used to treat thyroid disease, and it is also a naturally occurring thyroid hormone metabolite present in picomolar quantities in the serum of healthy humans [38]. Thyroid hormone ligands present in abnormally high concentrations pose a substantial health risk, prompting repeated FDA warnings against unprescribed dietary supplementation [38]. Biosensors detecting thyroid hormone ligands could help maintain the delicate hormone balance in a pharmacological setting and could preemptively identify toxic levels of interfering compounds to prevent inadvertent exposure.

## 2. Materials and Methods

### 2.1. Engineering and Design of the Biosensor Constructs

The biosensor constructs presented in this work were engineered based on our previously reported constructs [9,11,31] and *E. coli* β-galactosidase (β-gal) gene variations [26,28,30]. From N-terminus to C-terminus, the full-length β-gal construct gene contains: a maltose binding protein domain, an N-terminal intein, the human thyroid receptor beta (hTRβ), a C-terminal intein, and the *E. coli* β-gal. The β-gal-α construct is identical to the full-length β-gal construct except for the P67 residue of β-gal, which was mutated to be a stop codon so that the reporter protein is a truncated fragment—referred to as the alpha fragment—of β-gal. The split β-gal-α construct is similar to the β-gal-α construct but it does not include an N-terminal maltose binding fusion protein. Instead, it has an N-terminal portion of the alpha fragment which splices with a C-terminal alpha fragment upon ligand-induced conformational changes. 

The reporter sequences used in this study for the β-gal complementation of the split reporter proteins are based on the principle of alpha complementation that has been widely adopted in bioengineering applications such as a blue-white screening [25]. The use of β-gal-α as a strategically small reporter protein for a rapid cell-free biosensor was carried out similarly to previously reported methods [28], with the following specifications and modifications. An alpha donor fragment is expressed in the cell free system for enzyme complementation and activity [28,29]. In this study, the XL1-Blue cell extract contains the omega fragment of β-galactosidase to complement the donor alpha fragment synthesized in situ. 

The alpha fragment genes for the β-gal-α and split β-gal-α constructs are typically designed to include the first 40 residues of the N-terminal portion of β-gal, which are important for effective complementation [39], though longer fragments of 90 or more residues are commonly used [40], including in our prior work, where we used the pUC18 alpha fragment that is originally 107 amino acids in length [24]. For this work, an alpha fragment of 66 amino acids was chosen to avoid smaller minimal sequences that would be more subject to degradation than a larger fragment [41]. Thus, the β-gal residue P67 was mutated into a stop codon for a truncation of β-gal so that the protein functions as the alpha fragment of the β-gal-α construct, which becomes an active reporter after complementation with the omega fragment [40]. 

Spliced fragments of β-gal-α have been described in the literature [42] and various configurations were considered for the split β-gal-α construct designed in this work. The construct contains a spliced variant that was designed based on the principles of intein splicing that require a C-terminal extein (methionine or cysteine/serine) which forms a branched intermediate, which then leads to a spliced product connecting the C-terminal and N-terminal portions [43], as has been demonstrated with the T4 thymidylate synthase enzyme [24]. Accordingly, a *lacZ* alpha gene sequence, this time based on that of the pCM62 vector [44], was designed for splicing by splitting it in two fragments (Figure S3). Notably, the construct DNA and protein sequences for the full-length β-gal construct and the β-gal-α construct are the same except for the stop codon at position P67 of the β-gal protein, while the splicing β-gal-α construct is based on a different alpha fragment. The complete amino acid sequences for the biosensor constructs are included in the Supplementary Materials. 

DNA sequences encoding the biosensor constructs were cloned into a T7 expression plasmid. Purified plasmid was obtained by the Qiagen (Valencia, CA, USA) maxi kit according to the manufacturer’s instructions. 

The DNA for the biosensor construct using the β-gal-α reporter was created using Q5^®^ DNA Polymerase (NEB, Ipswich, MA, USA) to insert the stop codon within the *lacZ* gene of the full-length β-gal biosensor construct, with primers designed as reported in the literature [45]. 

### 2.2. Developing the XL1-Blue CFPS Platform

XL1-Blue cell extract was created using the following protocol: XL1-Blue competent cells (Agilent Technologies, Santa Clara, CA, USA) were used to inoculate 5 mL overnight culture of LB which was added to 100 mL 2×YT media and incubated at 37 °C and 280 rpm until a OD_600_ of ~2 when the 100 mL were transferred to 900 mL 2×YT. To induce β-gal omega fragment expression, IPTG was added to a final media concentration of 1 mM at OD_600_ of ~0.5 and cells were harvested by centrifugation at 6000 RCF for 10 min. Cells were homogenized at 20,000 psi with an Avestin Emulsiflex B15 (Avestin, Ottawa, ON, Canada). The resulting lysate was clarified by centrifugation at 12,000 RCF for 30 min and the supernatant, referred to as cell extract, was flash frozen and stored at −80 °C.

For XL1-Blue CFPS reactions requiring exogenously added T7 RNA Polymerase, T7 RNAP was expressed in *E. coli* BL21 DE3 STAR^TM^ cells with a pHT-T7RNAP-HIS plasmid and purified with the Akta Start Protein Purification System (GE Healthcare Bio-Sciences, Uppsala, Sweden) according to the manufacturer’s instructions. The purified formulation was added to CFPS reactions at an optimized concentration.

### 2.3. CFPS Reactions and Thyroid Ligand Assays

Cell-free protein synthesis (CFPS) reactions were conducted according to previously described methods [9,46,47] for PANOxSP reaction conditions with the following specifications. Amounts of 70 μL of CFPS reactions were assembled in 2 mL microcentrifuge tubes. The reactions were composed of 25 or 30% *v/v* XL1-Blue cell extract, 12 nM plasmid DNA, 0–20 mM magnesium glutamate (concentration adjusted for optimal protein yield), 1 mM 1,4-diaminobutane, 1.5 mM spermidine, 40 mM phosphoenolpyruvate, 10 mM ammonium glutamate, 175 mM potassium glutamate, 2.7 mM potassium oxalate, 0.33 mM nicotinamide adenine dinucleotide, 0.27 mM coenzyme A, 1.2 mM adenosine triphosphate (ATP), 0.86 mM cytidine triphosphate (CTP), 0.86 mM guanosine triphosphate (GTP), 0.86 mM uridine triphosphate (UTP), 0.17 mM folinic acid, 2 mM each of the amino acids (alanine, arginine, asparagine, aspartic acid, cysteine, glutamic acid, glutamine, glycine, histidine, isoleucine, leucine, lysine, methionine, phenylalanine, proline, serine, threonine, tryptophan, tyrosine, and valine), and an optimized volume fraction of purified T7 RNAP. 3,3′,5-triiodothyroacetic acid (TRIAC) was dissolved in dimethyl sulfoxide (DMSO) and, for consistency, all CFPS reactions were adjusted to have 5 volume percent DMSO regardless of the TRIAC concentration. CFPS reactions were performed at various concentrations of TRIAC, and *n* = 2 or 3 beta-galactosidase reactions were executed for each CFPS reaction condition. CFPS reactions were conducted at 30 °C for 4 h for the β-gal-α and split β-gal-α biosensors, or a 5.5 h reaction for the full-length β-gal construct, with shaking at 280 or 120 rpm, except sfGFP was synthesized at 37 °C for 3 h. Total and soluble protein expression yields were obtained using C14 Leucine (Moravek Inc., Brea, CA, USA) as described previously [48,49]. Briefly, CFPS samples were pipetted onto filter paper and then TCA precipitation of protein onto filter paper was followed by washing the papers in 5% ice cold TCA for 15 min, which was repeated 3 times. The radioactivity in the precipitated proteins was measured with a liquid scintillation counter. BL21 STAR^TM^ DE3 CFPS reactions were performed at 30 °C and 280 rpm for 4.5 h. BL21 cell extract was prepared as previously described [21].

CFPS reaction products were assessed for ligand-dependent activity with β-galactosidase enzymatic reactions of 100 or 150 μL. In addition to the CFPS sample, the reactions contained 9 mM ONPG in Z Buffer (60 mM Na_2_HPO_4_, 40 mM NaH_2_PO_4_, 1 mM MgCl_2_, 10 mM KCl, 2 mM DTT, pH 7.0) [8]. Activity assays were carried out at 37 °C in a SynergyMX plate reader (BioTek, Winooski, VT, USA) measuring the OD_420_ every 2 min for 30 min. Each data set was normalized to its highest observed OD_420_. To enhance the clarity of the ligand-dependent assay results, the cell-free reaction products for split β-gal-α had a 75-fold final dilution factor. The reaction products for full-length β-gal were centrifuged at 16,000 RCF for 10 min and diluted 15-fold in the assay. The reaction products for β-gal-α were centrifuged at 13,000 RCF for 10 min and diluted 13-fold in the assay.

### 2.4. Biosensor Reagent Cost Calculations

The cost analysis for the reagents required for the biosensors discussed in this work were computed assuming laboratory-scale unit prices. The cell-free protein synthesis basic reagents were valued at USD 4/mL [15] which incurs a USD 0.28 cost for a 0.070 mL reaction used in this work. This price is equivalent regardless of which construct and reporter enzyme (β-lac, β-gal, or variations thereof) are synthesized. The substrates o-nitrophenyl-β-D-galactopyranoside (ONPG) and nitrocefin could be purchased from Cayman Chemical for USD 13.44 per gram and USD 20,640 per gram, respectively, at the time this article was published. The substrate concentrations used to compute the assay costs were 2.81 mg/mL and 0.103 mg/mL [9], respectively, for ONPG and nitrocefin. Both assay systems were assumed to use the same 0.2 mL assay volume reported previously [9]. The assay costs were therefore USD 0.0076 and USD 0.43 for ONPG and nitrocefin, respectively, and each assay price is added to the USD 0.28 CFPS cost to derive the final biosensor reagent cost. The purpose of the cost analysis included in this work is to compare the costs of the two different biosensor substrate systems. Other notable cost considerations, such as labor cost, business operating cost, and scale-up unit price reduction, were not included in this cost analysis.

## 3. Results and Discussion

Previously, we engineered chimeric fusion protein sensors capable of detecting the potential endocrine disruptors that interact with the human thyroid receptor [9,10,11,31]. This sensor operates at the protein folding level, where the reporter domain is active when the linked human thyroid receptor domain interacts with the ligand during protein folding. To detect during protein folding, the fusion protein must be synthesized in the presence of the sample which makes the cell-free protein synthesis (CFPS) system an ideal sensing platform. Combining observations from in vivo and in vitro studies, this protein construct has successfully employed two reporter proteins: beta-lactamase (β-lac) and thymidylate synthase (TS), including a splicing version of TS [10,24,31]. It is interesting to note that, while the TS enzyme is active as a dimer, and the β-lac enzyme is monomerically active, the thyroid receptor biosensor construct creates a hormone-dependent readout with both reporters. Additional work reports evidence of the ligand-induced cleaving of the construct reporter enzyme domain which helps to enable the formation of reporter protein complexes [10]. This finding suggests that alternative reporter enzymes requiring tetramerization, complementation, and/or splicing are viable reporter candidates for biosensor construction. β-galactosidase (β-gal) is a popular tetrameric reporter protein due to its robust and rapid catalytic activity. One study reportedly measured the catalytic activity of one tetrameric complex to be 38,500 catalyzed reactions per minute [40]. Furthermore, β-gal can utilize colorimetric substrates, which is particularly useful for the simple visualization of enzymatic activity [30]. In this work, the biosensor is reengineered with β-gal reporter enzymes to enable the use of cost-effective colorimetric substrates such as ONPG used in this work.

Indeed, we report a 60% reduction in sensing cost by reengineering a chimeric fusion protein biosensor to include either the full-length β-gal, an alpha fragment of β-gal (β-gal-α), or a splicing alpha fragment of β-gal (split β-gal-α) as the reporter enzyme in order to utilize more cost-effective substrates than nitrocefin. The crude *E. coli* lysate, prepared in house for biosensing reactions, is estimated to cost USD 4/mL at the time of writing this article [15]. For the 0.070 mL reactions used in this work, the expected cost of a single test replicate in a reaction tube would be USD 0.28 and then an enzymatic assay follows. Previous studies using nitrocefin assay substrate require USD 0.43 for a 0.2 mL assay reaction, but the ONPG substrate utilized in this work reduces the assay substrate costs by 98% to USD 0.0076 which reduces the total biosensor cost by 60% (Figure S1). As part of future work, the CFPS reaction size could be scaled down to further reduce the biosensor cost. Other studies have explored paper-based options for reactions as small as 0.002 mL [23], which could decrease the USD 0.28 CFPS cost down to USD 0.008 per reaction. However, the scope of this work focuses on redesigning the biosensor constructs to reduce the assay substrate cost, which is the largest cost burden of the previously reported biosensor [9].

A cell-free biosensing system utilizing β-gal-α as a reporter enzyme requires the following: (1) a high-yielding cell extract without background β-gal activity and (2) a source of β-gal omega fragment. Various microbes, especially *E. coli* strains, have been engineered for customized CFPS applications [50,51], including biosensing [26,52]. The *E. coli* XL1-Blue strain retains a mutation in the *lacZ* gene which causes the strain to express the β-gal omega fragment instead of the full-length β-gal enzyme, satisfying both requirements. Accordingly, XL1-Blue cells were cultured and processed according to protocols that have been developed for the production of low-cost crude cell extracts. The resultant cell lysate indeed functions as expected, with low residual β-gal activity in the absence of a source of alpha fragment and high β-gal activity when the alpha fragment is added to the system or produced in situ during cell-free protein synthesis. Thus, the XL1-Blue cell extract is a valuable addition to the CFPS platform. 

**Figure 2:**
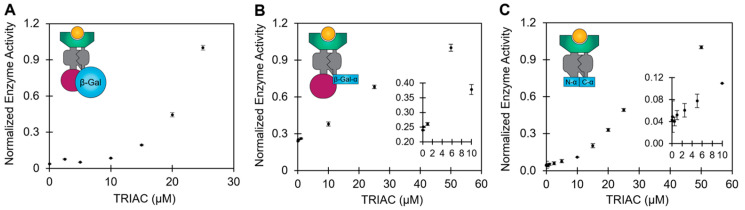
Normalized enzymatic activity of the fusion protein biosensors engineered in this work in the presence of increasing concentrations of TRIAC. A visual representation of each protein construct portrays the respective protein domains, including a maltose binding protein (magenta), intein (grey), human thyroid receptor β (green), intein (grey), and a β-gal reporter (blue). The TRIAC ligand is also represented (orange). (**A**) Full-length beta galactosidase (β-gal) biosensor construct, (**B**) β-gal-α biosensor construct, and (**C**) split β-gal-α biosensor construct. Assay data points were obtained after 30 min of the enzymatic assay, which was preceded by a 4 h CFPS reaction for the β-gal-α and split β-gal-α biosensors, or a 5.5 h reaction for the full-length β-gal construct. Data are the average of *n* = 2 replicates and the error bars are the standard deviations.

The three constructs have differing structural domains yet all three engineered biosensor constructs successfully responded to human thyroid receptor ligand in a concentration-dependent manner (Figure 2). These encouraging results with the engineered biosensor constructs further demonstrate the versatility and modularity of the biosensor scaffold and its potential for diverse use in gene circuits and biosensing applications. Each fusion protein construct has the human thyroid receptor β domain for ligand interaction, flanked by intein domains, a maltose binding protein, and a reporter protein (Figure 2A,B). The split β-gal-α does not include an N-terminal maltose binding fusion protein; instead, it has an N-terminal portion of the alpha fragment which splices with a C-terminal alpha fragment upon ligand-induced conformational changes (Figure 2C).

For reporter activity, the β-gal reporter requires tetramerization while the β-gal-α requires complementation with the omega fragment prior to tetramerization. The split β-gal-α reporter requires splicing, complementation, and tetramerization. Thus, the full-length β-gal has the simplest formation of an active complex, but the large size of full-length β-gal is more cumbersome and slow for a rapid assay than a much smaller alpha fragment [29]. The amino acid sequence lengths for the constructs are 1842, 884, and 536 for the full-length β-gal, β-gal-α, and split β-gal-α, respectively (Figure S3). The β-gal-α and split β-gal-α reporters are recommended as the most promising candidates for future work toward a rapid assay due to much smaller reporter sizes than the full-length β-gal, though optimizing assay speed is beyond the scope of this work. The scope of this work is to demonstrate that all three constructs successfully detect thyroid disruptor ligands with a 60% cost reduction in the biosensor.

**Figure 3:**
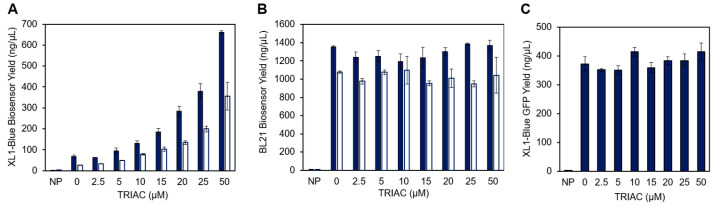
Cell-free protein synthesis yields of the full-length β-gal biosensor or sfGFP in the presence of increasing concentrations of TRIAC. Blue bars denote total protein yield, white bars denote soluble protein yield, and error bars represent the standard deviation of *n* = 2 or 3 replicates. NP indicates a no plasmid reaction used as a negative control. (**A**) Cell-free protein synthesis yield of the full-length β-gal biosensor with XL1-Blue cell extract. (**B**) Cell-free protein synthesis yield of the full-length β-gal biosensor with BL21 DE3 cell extract. (**C**) Cell-free protein synthesis yield of superfolder green fluorescent protein (sfGFP) with XL1-Blue cell extract.

The reengineered constructs demonstrate ligand-dependent enzymatic activity in XL1-Blue extract as expected (Figure 2), but in contrast to our previous results [9], the overall CFPS production of the new constructs was ligand-dependent (Figure 3A and Figure S2A,B). In a parallel experiment, the β-gal construct was synthesized in BL21 CFPS and ligand-independent protein synthesis was observed (Figure 3B), which corroborates prior work [9]. Furthermore, the XL1-Blue cell-free protein synthesis of sfGFP in the presence of TRIAC did not reveal a strong ligand-dependent yield (Figure 3C), which rules out nonspecific ligand interactions with the XL1-Blue cell extract. Thus, the TRIAC-dependent CFPS protein yield is specific to the production of a hTRβ biosensor construct in the XL1-Blue extract. Prior to the commercialization of the new biosensor constructs presented in this work, extensive validation work will also need to rule out nonspecific interactions with diverse sample matrices to screen for false positives or unforeseen interference. Future validation work is especially important for EDC screening and the biosensors used to guide the administration of pharmaceutical hormone intervention. However, prior work demonstrated that blood and raw sewage are compatible with the CFPS biosensor, and no false positive or false negative responses were observed [9,11], which is encouraging for future commercial validation studies. 

Given the observation of ligand-dependent protein synthesis in the XL1-Blue extract but not the BL21 extract, we hypothesize that protease degradation may be responsible for the variation in CFPS protein yield based on the following information: (1) the conformational stability of the biosensor protein construct increases upon ligand binding [10]; (2) BL21 DE3 strains are deficient of the Lon and OmpT protease activity [53,54,55], which are highly active on partially folded proteins [56]; and (3) protein yields were determined by scintillation counting after TCA precipitation which precipitates proteins but not degraded peptide fragments <0.5 kDa [57]. However, the most significant finding of this work is the successful development of three reengineered thyroid receptor ligand biosensors with unique β-gal reporter domains (Figure 2). These sensors effectively reduce the overall cost by 60%.

The 95% cost saving from utilizing the ONPG substrate does come at a minor tradeoff cost of producing T7 RNA Polymerase in a separate step to supplement the XL1-Blue extract. Fortunately, T7 production methods for use in a crude-extract expression system can be streamlined [58] to a protocol resembling cell extract production, which represents a small fraction of the USD 0.28 CFPS cost [15], thus the cumulative engineering adjustments in this work do provide net cost savings of approximately 60% for the biosensor. Furthermore, other cell lines could be engineered to make cell extracts, such as a BL21 DE3 strain with the same *lacZ* gene manipulation that the XL1-Blue strain has, which could result in a cell line that produces T7 RNA Polymerase and a *lacZ* omega fragment. However, this work reports the use of XL1-Blue cell extract in cell-free protein synthesis by using separately purified T7 RNA Polymerase to utilize the popular T7 expression system and the low-cost β-gal colorimetric substrates for ligand detection.

## 4. Conclusions

Thyroid receptor ligands have impactful toxicologic and pharmacologic implications for healthy living and the mitigation of severe health maladies including cancer, metabolic disorders, and neurodevelopment abnormalities. Previously, we reported on thyroid ligand detection capability with cell-free protein synthesis of a chimeric fusion protein containing the hTRβ receptor activator and the β-lac reporter. In this work, we report a 60% reduction in sensing cost by reengineering a chimeric fusion protein biosensor to include one of three different β-gal reporter systems (full-length β-gal, β-gal-α, or split β-gal-α). These biosensor constructs are deployed using *E. coli* XL1-Blue cell extract to avoid the β-gal background activity abundant in BL21 cell extract and to facilitate split β-gal reporter activity to detect human thyroid receptor ligands. These advancements in reengineering the biosensor with alternative reporter proteins further demonstrate the versatility and modularity of the biosensor scaffold and its potential for diverse use in gene circuits and biosensing applications. Furthermore, the cost-reduction measures presented in this work streamline a promising biosensing platform for low-cost high-throughput screening and the potentially portable detection of human thyroid receptor ligands to help prevent inadvertent exposure to endocrine disrupting compounds.

## Data Availability

The data presented in this study are available within the article and supplementary information.

## Supplementary Materials

The following supporting information can be downloaded at: https://www.mdpi.com/article/10.3390/life13101972/s1, Figure S1: Cost analysis of select colorimetric substrates for β-lactamase (β-lac) and β-galactosidase (β-gal) reporter enzymes, Figure S2: XL1-Blue cell-free protein synthesis of biosensor constructs, Figure S3: Amino acid sequences of the biosensor constructs presented in this work.
