# Supplementary material for: Streamlining the Detection of Human Thyroid Receptor Ligand Interactions with XL1-Blue Cell-Free Protein Synthesis and Beta-Galactosidase Fusion Protein Biosensors
Source: Life (Basel). 2023 Sep 27;13(10):1972. doi: 10.3390/life13101972 (PMC10608756; doi:10.3390/life13101972)
Supplement: Supplementary file 1 [file life-13-01972-s001.zip › life-2599935-supplementary.pdf]

life

MDPI

Article: Supplementary Information

Streamlining the Detection of Human Thyroid Receptor Ligand Interactions with XL1-Blue Cell-free Protein Synthesis and Beta-galactosidase Fusion Protein Biosensors

J. Porter Hunt [1], Tyler J. Free [1], Jackelyn Galiardi [2], Kevin M. Watt [3], David W. Wood [2], and Bradley C. Bundy [1,*]

[1] Department of Chemical Engineering, Brigham Young University, Provo, UT 84602, USA
[2] Department of Chemical and Biomolecular Engineering, the Ohio State University, Columbus, OH 43210, USA
[3] Department of Pediatrics, University of Utah, Salt Lake City, UT 84108, USA
* Correspondence: bundy@byu.edu

Figure S1: Cost analysis of colorimetric substrates for β-lactamase (β-lac) and β-galactosidase (β-gal) reporter enzymes. The β-gal substrate o-nitrophenyl-β-D-galactopyranoside (ONPG) is compared with the β-lac substrate nitrocefin. The cost per assay is computed with vendor pricing available at the time this article was published and the substrate concentrations reported in the respective references. The assay volume is assumed to be 0.2 mL.

Reporter Enzyme	Substrate	$ per assay	Reference
β-Gal	ONPG	0.0076	This work
β-Lac	Nitrocefin	0.4252	[9],[11]

Figure S2: XL1-Blue cell-free protein synthesis of biosensor constructs. Blue bars denote total protein yield, white bars denote soluble protein yield, and error bars represent the standard deviation of $n = 2$ replicates. NP indicates a no plasmid reaction used as a negative control. A) Cell-free protein synthesis yield of β-gal-α biosensor at different TRIAC concentrations. B) Cell-free protein synthesis yield of split β-gal-α biosensor at different TRIAC concentrations.

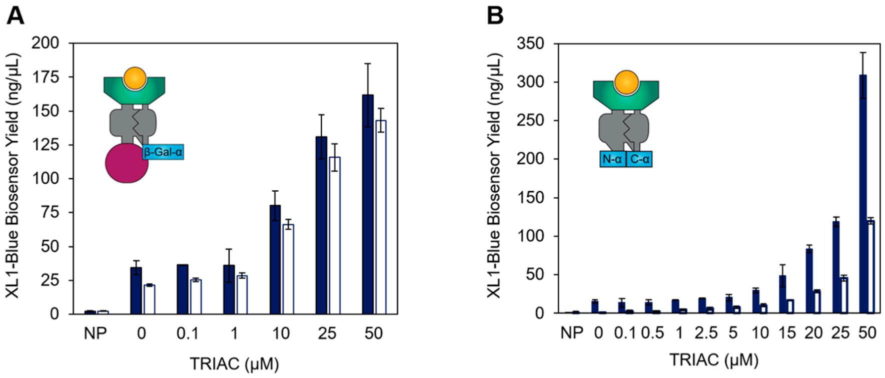

Figure S3: Amino acid sequences and visual representations of the three biosensor constructs presented in this work. The sequences are annotated with the following colors to represent the domains of the fusion protein biosensor construct:

β-gal Biosensor Construct: Maltose binding protein (magenta), intein (grey), human thyroid receptor β (green), intein (grey), β-gal reporter (blue). The TRIAC ligand is also shown (orange).

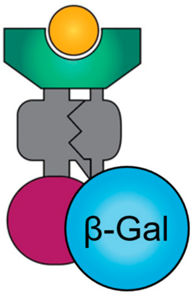

MKIEEGKLVIWINGDKGYNGLAEVGKKFEKDTGIKVTVEHPDKLEEKFPQVAATGDGPDIIFWAHD

RFGGYAQSGLLAEITPDKAFQDKLYPFTWDAVRYNGKLIAYPIAVEALSLIYNKDLLPNPPKTWEEI

PALDKELKAKGKSALMFNLQEPYFTWPLIAADGGYAFKYENGKYDIKDVGVDNAGAKAGLTFLV

DLIKNKHMNADTDYSIAEAAFNKGETAMTINGPWAWSNIDTSKVNYGVTVLPTFKGQPSKPFVGV

LSAGINAASPNKELAKEFLENYLLTDEGLEAVNKDKPLGAVALKSYEEELAKDPRIAATMENAQKG

EIMPNIPQMSAFWYAVRTAVINAASGRQTVDEALKDAQTNSSSNNNNNNNNNNLGIEGRISEFALA

EGTRIFDPVTGTTHRIEDVVGGRKPIHVVAAAKDGTLHARPVVSWFDQGTRDVIGLRIAGGAIVWA

TPDHKVLTEYGWRAAGELRKGDRVAQPRRFDGFGDSAPIPAELQKSIGHKPEPTDEEWELIKTVTE

AHVATNAQGSHWKQKRKFLPEDIGQAPIVNAPEGGKVDLEAFSHFTKIITPAITRVVDFAKKLPMFC

ELPCEDQIILLKGCCMEIMSLRAAVRYDPESETLTLNGEMAVTRGQLKNGGLGVVSDAIFDLGMSLS

SFNLDDTEVALLQAVLLMSSDRPGLACVERIEKYQDSFLLAFEHYINYRKHHVTHFWPKLLMKVTD

LRMIGACHASRFLHMKVECPTELFPPLFLEVFEDRVQALADALDDKFLHDMLAEELRYSVIREVLPT

RRARTFDLEVEELHTLVAEGVVVHNMTMITDSLAVVLQRRDWENPGVTQLNRLAAHPPFASWRNS

EEARTDRPSQQLRSLNGEWRFAWFPAPEAVPESWLECDLPEADTVVVPSNWQMHGYDAPIYTNVT

YPITVNPPFVPTENPTGCYSLTFNVDESWLQEGQTRIIFDGVNSAFHLWCNGRWVGYGQDSRLPSEF

DLSAFLRAGENRLAVMVLRWSDGSYLEDQDMWRMSGIFRDVSLLHKPTTQISDFHVATRFNDDFS

RAVLEAEVQMCGELRDYLRVTVSLWQGETQVASGTAPFGGEIIDERGGYADRVTLRLNVENPKLW

SAEIPNLYRAVVELHTADGTLIEAEACDVGFREVRIENGLLLLNGKPLLIRGVNRHEHHPLHGQVMD

EQTMVQDILLMKQNNFNAVRCSHYPNHPLWYTLCDRYGLYVVDEANIETHGMVPMNRLTDDPR

WLPAMSERVTRMVQRDRNHPSVIIWSLGNESGHGANHDALYRWIKSVDPSRPVQYEGGGADTTAT

DIICPMYARVDEDQPFPAVPKWSIKKWLSLPGETRPLILCEYAHAMGNSLGGFAKYWQAFRQYPRL

QGGFVWDWVDQSLIKYDENGNPWSAYGGDFGDTPNDRQFCMNGLVFADRTPHPALTEAKHQQQ

FFQFRLSGQTIEVTSEYLFRHSDNELLHWMVALDGKPLASGEVPLDVAPQGKQLIELPELPQPESAG

QLWLTVRVVQPNATAWSEAGHISAWQQWRLAENLSVTLPAASHAIPHLTTSEMDFCIELGNKRWQ

FNRQSGFLSQMWIGDKKQLLTPLRDQFTRAPLDNDIGVSEATRIDPNAWVERWKAAGHYQAEAAL

LQCTADTLADAVLITTAHAWQHQGKTLFISRKTYRIDGSGQMAITVDVEVASDTPHPARIGLNCQL

AQVAERVNWLGLGPQENYPDRLTAACFDRWDLPLSDMYTPYVFPSENGLRCGTRELNYGPHQWR

GDFQFNISRYSQQQLMETSHRHLLHAEEGTWLNIDGFHMGIGGDDSWSPSVSAEFQLSAGRYHYQL

VWCQK

β-gal-*α* biosensor construct**:** Maltose binding protein (magenta), intein (grey), human thyroid receptor β (green), intein (grey), β-gal-*α* reporter (blue)

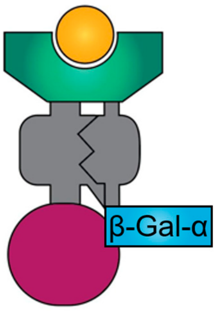

MKIEEGKLVIWINGDKGYNGLAEVGKKFEKDTGIKVTVEHPDKLEEKFPQVAATGDGPDIIFWAHD
RFGGYAQSGLLAEITPDKAFQDKLYPFTWDAVRYNGKLIAYPIAVEALSLIYNKDLLPNPPKTWEEI
PALDKELKAKGKSALMFNLQEPYFTWPLIAADGGYAFKYENGKYDIKDVGVDNAGAKAGLTFLV
DLIKNKHMNADTDYSIAEAAFNKGETAMTINGPWAWSNIDTSKVNYGVTVLPTFKGQPSKPFVGV
LSAGINAASPNKELAKEFLENYLLTDEGLEAVNKDKPLGAVALKSYEEELAKDPRIAATMENAQKG
EIMPNIPQMSAFWYAVRTAVINAASGRQTVDEALKDAQTNSSSNNNNNNNNNNLGIEGRISEFALA
EGTRIFDPVTGTTHRIEDVVGGRKPIHVVAAAKDGTLHARPVVSWFDQGTRDVIGLRIAGGAIVWA
TPDHKVLTEYGWRAAGELRKGDRVAQPRRFDGFGDSAPIPAELQKSIGHKPEPTDEEWELIKTVTE
AHVATNAQGSHWKQKRKFLPEDIGQAPIVNAPEGGKVDLEAFSHFTKIITPAITRVVDFAKKLPMFC
ELPCEDQIILLKGCCMEIMSLRAAVRYDPESETLTLNGEMAVTRGQLKNGGLGVVSDAIFDLGMSLS
SFNLDDTEVALLQAVLLMSSDRPGLACVERIEKYQDSFLLAFEHYINYRKHHVTHFWPKLLMKVTD
LRMIGACHASRFLHMKVECPTELFPPLFLEVFEDRVQALADALDDKFLHDMLAEELRYSVIREVLPT
RRARTFDLEVEELHTLVAEGVVVHNMTMITDSLAVVLQRRDWENPGVTQLNRLAAHPPFASWRNS
EEARTDRPSQQLRSLNGEWRFAWFPA

Split β-gal-*α* biosensor construct: N-terminal splice fragment of β-gal-*α* (blue), intein (grey), human thyroid receptor β (green), intein (grey), C-terminal splice fragment of β-gal-*α* reporter (blue)

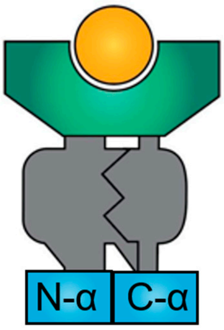

MTMITPSLHACLAEGTRIFDPVTGTTHRIEDVVDGRKPIHVVAAAKDGTLHARPVVSWFDQGTRDV
IGLRIAGGAILWATPDHKVLTEYGWRAAGELRKGDRVAQPRRFDGFGDSAPIPARELQKSIGHKPEP
TDEEWELIKTVTEAHVATNAQGSHWKQKRKFLPEDIGQAPIVNAPEGGKVDLEAFSHFTKIITPAITR
VVDFAKKLPMFCELPCEDQIILLKGCCMEIMSLRAAVRYDPESETLTLNGEMAVTRGQLKNGGLGV
VSDAIFDLGMSLSSFNLDDTEVALLQAVLLMSSDRPGLACVERIEKYQDSFLLAFEHYINYRKHHVT
HFWPKLLMKVTDLRMIGACHASRFLHMKVECPTELFPPLFLEVFEDARVQALADALDDKFLHDML
AEELRYSVIREVLPTRRARTFDLEVEELHTLVAEGVVVHNCRSTLEDPRVPSSNSLAVVLQRRDWE
NPGVTQLNRLAAHPPFASWRNSEEARTDRPSQQLRSLNGEWRLMRYFLLTHLCGISHRIWCTLSTIC
SDAA
